# Patients’ needs, expectations & concerns regarding clear aligner treatment

**DOI:** 10.1007/s00784-026-06903-x

**Published:** 2026-05-29

**Authors:** Michael Nemec, Linda Schwarz, Leonie Schaller, Karin Schweiger, Stefan Lettner, Bärbl Reistenhofer, Kristina Bertl, Erwin Jonke

**Affiliations:** 1 Division of Orthodontics, University Clinic of Dentistry, Medical University of Vienna, Sensengasse 2a, Vienna, 1090 Austria; 2 Department of Orthodontics, Dental Clinic, Faculty of Medicine, Sigmund Freud Private University, Freudplatz 3, Vienna, 1020 Austria; 3 Private practice, Dr.-Natzler-Gasse 8, Perchtoldsdorf, 2380 Austria; 4 Core Facility Hard Tissue and Biomaterials Research, University Clinic of Dentistry, Medical University of Vienna, Sensengasse 2a, Vienna, 1090 Austria; 5 Private practice, Rooseveltplatz 12, Vienna, 1090 Austria; 6 Department of Periodontology, Dental Clinic, Faculty of Medicine, Sigmund Freud University Vienna, Freudplatz 3, Vienna, 1020 Austria; 7 Department of Periodontology, Blekinge Hospital, Byggnad 13 Plan 8, Karlskrona, 371 85 Sweden

**Keywords:** Clear aligner, Orthodontic treatment, Patients’ expectations, Questionnaire

## Abstract

**Objective:**

Patients aiming for clear aligner therapy (CAT) have a distinct expectation regarding their orthodontic treatment. The aim of this prospective cohort study was to increase our knowledge on these specific patients’ needs, expectations, and concerns regarding CAT.

**Methods:**

Patients, who were about to start CAT, were asked to answer both, a 32-item questionnaire addressing expectations regarding CAT, rated on a 10-point visual analogue scale, and the Oral Health Impact Profile (OHIP-14) questionnaire. In addition, demographic and clinical data were collected.

**Results:**

A total of 82 patients participated (mean age 35 ± 12 years; 74.4% female). Patients strongly expected CAT to treat their malocclusion as effectively as orthodontic brackets (9.5 ± 0.9 points) and they would seek a second opinion if they were told treatment with CAT would not be possible (7.2 ± 3.3 points). In addition, patients appeared not willing to compromise on the planned treatment outcome, even if it would decrease treatment time (2.1 ± 2.3 points) or costs (2.4 ± 2.5 points). Further, they highly expected that the treatment result would endure for their lifetime (8.8 ± 2.1 points), they wanted to avoid visibility of orthodontic brackets (7.9 ± 2.8 points), prevent potential future problems (9.1 ± 1.5 points), and see a simulation of the expected treatment outcome (7.5 ± 2.9 points). The most important limitations with CAT expected by the participants were pain during treatment (7.7 ± 2.7 points) and restricted eating and drinking habits (7.3 ± 2.8 points).

**Conclusion:**

Patients considering CAT exhibit high expectations, particularly regarding treatment outcomes, aesthetics, and efficiency and they appear not willing to accept any compromise due to choosing CAT.

**Clinical relevance:**

Knowledge about these expectations can support our patient communication and thereby improve patients’ satisfaction with the treatment outcome.

**Supplementary Information:**

The online version contains supplementary material available at 10.1007/s00784-026-06903-x.

## Background

 Modern orthodontics is aiming to provide patients with a comfortable and pleasant treatment experience, which lead to a growing popularity of clear aligner therapy (CAT) over traditional fixed orthodontic appliances; in particular due to superior aesthetics, higher patient acceptance, reduced impact on oral health related quality of life (OHRQoL), and more simple oral hygiene during treatment [1–9]. More specifically, aligners are removable, which allows patients to maintain a good oral hygiene, thus reducing the risk of plaque accumulation and development of gingivitis compared to patients with braces [4, 10]. Further, aligners are appealing to patients prioritizing aesthetics and the invisibility of their orthodontic devices [3, 11, 12] for example, it has been shown that patients treated with aligners tend to report less embarrassment when laughing due to their orthodontic treatment [7]. In addition, aligners probably offer a more comfortable experience, with fewer incidences of mucosal lesions and irritations due to the lack of metal brackets and wires [5, 48].

Patients’ choice for either clear aligners or fixed appliances depends on various factors, including gender, age, and severity of malocclusion [12]. In addition, patients are often choosing orthodontic treatment due to aesthetic considerations [13, 14], which places new demands on everyday practice, especially in private clinics [15]. The majority of patients seeking CAT rejected fixed orthodontic treatment due to aesthetic concerns and the predominant source of their information about CAT was not coming from their general dentist (22%) or orthodontist (20%), but rather via media channels (41%) [12]. Altogether, understanding patients’ views and desires is essential to offer satisfactory treatment outcomes [16].

In this context, several studies have explored patient experiences during treatment with CAT (for overview see: [11]), but the existing literature on patient expectations prior to treatment initiation remains limited. For example, one retrospective study [17] relied on expectations documented in dental records rather than a structured assessment of the patients’ opinion, while other studies analyzed social media posts and therefore primarily publicly expressed attitudes [18, 19], surveyed general dental patients or laypeople not specifically pursuing orthodontic treatment [20, 21], or the data were already collected more than 20 years ago [22]. Therefore, the aim of this prospective cohort study was to increase our knowledge on the needs, expectations, and experiences of patients regarding CAT based on a prospectively distributed questionnaire prior to, during, and after CAT. In the present manuscript, the data collected prior to CAT initiation are reported, including, as a secondary aim, the assessment of any associations between patients’ expectations and patient-specific factors, such as gender, age, severity of malocclusion, and OHRQoL.

## Methodology

### Study design and patient recruitment

Participants of this prospective cohort study were recruited from patients scheduled to undergo CAT either in a private orthodontic practice in Vienna or in the Division of Orthodontics of the University Dental Clinic of Vienna (Medical University of Vienna) between September 2019 and July 2022 with either Invisalign “Comprehensive Package” or “Lite Package”. Patients with former Invisalign “i7 Package” treatments or “Express Package” were not included into the survey. Patients were informed about the study procedure, that participation in the study was voluntary, and all participants provided written informed consent. Patients scheduled for orthodontic treatment with clear aligners (Invisalign, Align Technology Inc., Tempe, Arizona) were eligible for inclusion, while patients with a history of previously completed aligner treatment, being < 18 years old, and/or undergoing combination therapy with fixed appliances were excluded. The study protocol was approved by the Ethics Committee of the Medical University of Vienna (EK-No. 1563/2019) and registered on clinicaltrials.gov (NCT04105491).

### Sample size calculation

Due to the pilot nature of this study and the fact that the main questionnaire has not been used previously, the sample size calculation was based on the OHIP and not on the primary outcome, i.e., the exact power for the primary outcome is unknown. Assuming approximate normality, a sample size of 66 was calculated to achieve standard errors small enough (i.e., 0.25) to reasonably base future confirmatory studies on the results. Accounting for a potential dropout rate of about 20%, the final sample size was set at 84 patients distributed between 2 centers.

### Data collection

Based on a non-validated questionnaire, patients’ needs, expectations, and concerns regarding CAT were assessed prior to treatment initiation. This questionnaire comprised 32 statements regarding CAT (see Tables 2, 3, 4, 5 and 6), which were constructed within a group of experienced orthodontists. The patients marked their agreement for each statement on a 50 mm visual analogue scale (VAS). These responses were measured and subsequently converted to a 0–10-point scale. The 32 statements were divided into the following five categories, based on a factor analysis: (1) Limitations (*n* = 9), (2) Second Opinion (*n* = 5), (3) Efficiency and Predictability (*n* = 6), (4) Willingness to Compromise (*n* = 4), and (5) Quality of Treatment (*n* = 8). The results were additionally classified into 3 categories according to the reported agreement using the following cut-off values: “mild” (< 3), “moderate” (3–7), and “strong” (> 7) agreement. Further, all participants answered the German OHIP-14 questionnaire [23] to assess OHRQoL. Responses were rated on a 5-point Likert scale, with 0 indicating “never” and 4 indicating “very often”. An OHIP simple count score was calculated as defined by El Osta et al. [24]. Items with the responses “always”, “often”, and “sometimes” were collapsed into one category and participants received for each question either 1 point if “sometimes” or more often was chosen or 0 points if less than “sometimes” was chosen. Then the sum of all questions was calculated with a maximum of 14 points. In addition, patients’ age, gender, educational degree, as well as the initial “Peer Assessment Rating” (PAR) index [25] were extracted from the questionnaire and clinical records. The PAR index is a numerical measurement of malocclusion severity and provides a standardised score that can be used to evaluate the outcome of orthodontic treatment.

### Statistical analysis

The statistical analysis was conducted using R version 4.4.3. Descriptive statistics included means and standard deviations. As an exploratory analysis without correction for multiple testing, two-sample *t*-tests were performed to test for different responses according to the subgroups (1) gender (male vs. female), (2) age (≤ 35 years vs. > 35 years), (3) initial PAR index (≤ 17 vs. > 17), and (4) initial OHIP simple count score (< 1 vs. ≥ 1). The cut-off value of 17 for the PAR index was chosen according to previous studies [26, 27]. Factor analyses with several types of rotations were tried but deemed too heterogenous for the purpose of this paper. The results of the factor analyses were only used to roughly group questionnaire items into smaller subgroups (i.e., category 1 to 5) for better readability. Cronbach’s alpha values are provided for overview for each category, which met an often used cut-off value of 0.7 for 3 out of 5 categories, while category 3 (Cronbach’s α of 0.64) and category 5 (Cronbach’s α of 0.38) were below this cut-off value [28].

## Results

### Study population

A total of 82 patients participated and completed the questionnaire prior to CAT initiation, with 52.7% having been treated at the university clinic and 46.3% at the private practice. The mean age of the participants was 35 ± 12 years. Most participants were female (74.4%) and 48.8% of the participants had attained a higher educational qualification. For details see Table 1.

**Table 1:** Demographic data of the patient sample

Number of participants (*n*)		82
Age (years; mean ± SD)		35 ± 12
Gender [female; n (%)]		61 (74.4)
Study centre [n (%)]	*University clinic*	44 (53.7)
	*Private practice*	38 (46.3)
PAR index	*Mean ± SD*	19 ± 10
	*≤ 17 [n (%)]*	42 (51.2)
	*> 17 [n (%)]*	38 (46.4)
	*Missing values*	2 (2.4)
OHIP score	*Mean ± SD*	5.9 ± 5.8
	*Simple count < 1 [n (%)]*	36 (43.9)
	*Simple count ≥ 1 [n (%)]*	46 (56.1)
Highest educational degree [n (%)]	*Compulsory school*	4 (4.9)
	*Apprenticeship*	12 (14.6)
	*A-levels*	24 (29.3)
	*Higher educational degree*	40 (48.8)
	*Missing values*	2 (2.4)

Abbreviations: *n* number, *OHIP* Oral Health Impact Profile, *PAR* peer assessment rating, *SD* standard deviation

### Patients’ expectations regarding limitations with CAT (Category 1, Table 2)

The most important limitations with CAT expected by the participants were pain that might occur during treatment (7.7 ± 2.7 points) and restricted eating and drinking habits (7.3 ± 2.8 points). In addition, it was highly important for the participants to avoid visibility of orthodontic brackets (7.9 ± 2.8 points). In contrast, patients expressed only moderate concerns that aligners might be perceived by other people (5.8 ± 3.2 points), about potential restrictions caused by using attachments (4.1 ± 3.1 points) and impairment of oral hygiene due to CAT (3.1 ± 2.9 points). The patients hardly expected any influence on their self-esteem (2.3 ± 2.5 points).

**Table 2:**
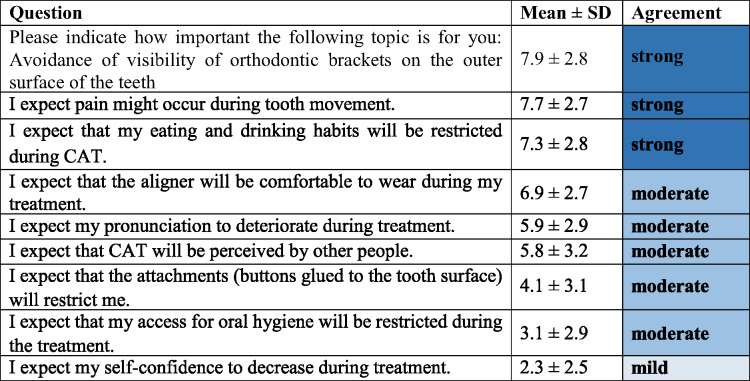
Patients’ expectations regarding limitations with CAT (Category 1, Cronbach’s α = 0.72)

*CAT* clear aligner therapy, *SD* standard deviation

### Patients’ expectations regarding second opinion for CAT (Category 2, Table 3)

Patients strongly agreed to seek a second opinion if they were told treatment with CAT were not possible (7.2 ± 3.3 points). There was moderate agreement to get a second opinion if CAT would only be possible in conjunction with tooth extraction (6.7 ± 3.6 points), interproximal enamel reduction (5.2 ± 3.6 points), or orthodontic brackets (6.1 ± 3.7 points).

**Table 3:**
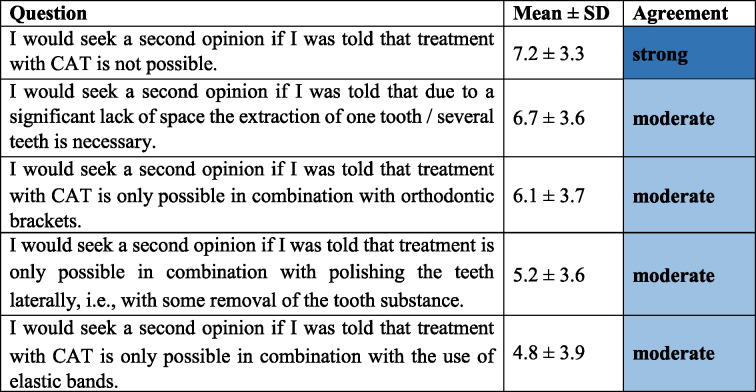
Patients’ expectations regarding seeking a second opinion for CAT (Category 2, Cronbach’s α = 0.78)

*CAT* clear aligner therapy, *SD* standard deviation

### Patients’ expectations regarding efficiency and predictability of CAT (Category 3, Table 4)

Efficiency and predictability were highly important to patients seeking CAT. In detail, they considered the predictability of treatment success as very important (8.6 ± 1.9 points), as well as time efficiency (7.7 ± 2.4 points), and the prevention of potential future problems (9.1 ± 1.5 points). Further, it was important to them to see a simulation of the expected treatment outcome (7.5 ± 2.9 points) as well as to receive a cost-effective treatment (7.1 ± 2.7 points).

**Table 4:**
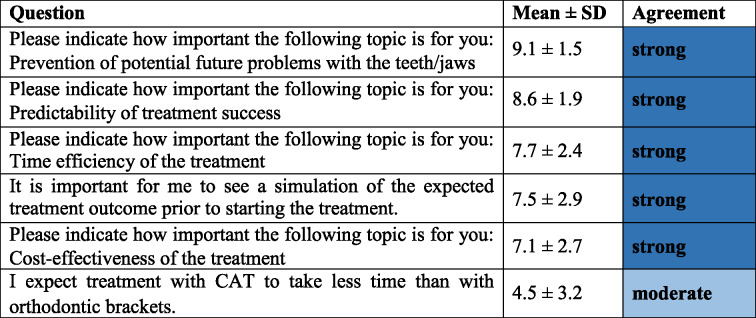
Patients’ expectations regarding efficiency and predictability of CAT (Category 3, Cronbach’s α = 0.64)

*CAT* clear aligner therapy, *SD* standard deviation

### Patients’ expectations regarding willingness to compromise with CAT (Category 4, Table 5)

Overall, patients appeared not willing to compromise on the planned treatment outcome. Specifically, they would not want to reduce treatment time (2.1 ± 2.3 points) and treatment costs (2.4 ± 2.5 points) if this would result in a slightly inferior improvement of the tooth position. Further, they would not deny the use of elastic bands (2.6 ± 2.8 points) or attachments on the tooth surface (2.4 ± 2.7 points) if this would limit the possibilities of tooth movement and thereby treatment outcome.

**Table 5:**
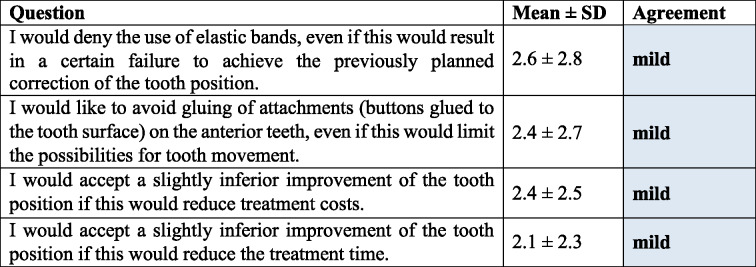
Patients’ expectations regarding willingness to compromise with CAT (Category 4, Cronbach’s α = 0.73)

*SD* standard deviation

### Patients’ expectations regarding quality of treatment with CAT (Category 5, Table 6)

Patients strongly expected CAT to treat their malocclusion as effectively as orthodontic brackets (9.5 ± 0.9 points). However, they only moderately agreed that CAT would treat their malocclusion better than orthodontic brackets (5.8 ± 3.2 points). Furthermore, patients had the expectation that the result of their treatment would endure for a period of at least five years (8.7 ± 2.9 points) or even for the duration of their lifetime (8.8 ± 2.1 points) and patients moderately agreed that their dentist should be present at each appointment during CAT (7.0 ± 3.0 points). However, patients did not expect their treatment to have a relevant effect on their job opportunities (2.4 ± 2.5 points).

**Table 6:**
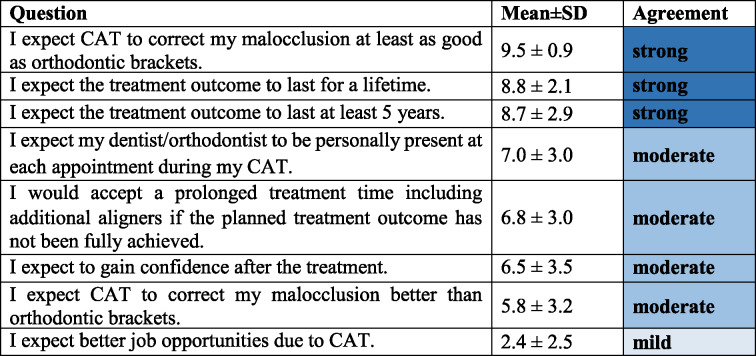
Patients’ expectations regarding quality of treatment with CAT (Category 5, Cronbach’s α = 0.38)

*CAT* clear aligner therapy, *SD* standard deviation

### Exploratory subgroup analyses

As an exploratory analysis without correction for multiple testing, patients’ responses were categorized and compared based on gender, age, initial PAR index, and OHIP simple count score but only the significant differences are summarized herein. All data derived from the subgroups are displayed in the Supplementary material (Tables 1, 2, 3, 4 and 5 S).

Female patients indicated significantly higher importance on the predictability of treatment success (8.9 ± 1.6 points vs. 7.7 ± 2.4 points, *p* = 0.037) and treatment time efficiency (8.1 ± 2.3 points vs. 6.8 ± 2.2 points, *p* = 0.036) compared to male patients. Further, male patients more likely denied the use of elastic bands during CAT compared to women, i.e., 3.7 ± 2.9 and 2.2 ± 2.7 points, respectively (*p* = 0.034). Finally, female patients agreed stronger on the statement that CAT would correct their malocclusion better than orthodontic brackets (6.2 ± 3.4 points vs. 4.5 ± 2.6 points, *p* = 0.022).

For patients > 35 years of age, the time efficiency was more relevant compared to younger patients (8.5 ± 1.8 points vs. 7.1 ± 2.5 points, *p* = 0.007). Further, patients > 35 years of age agreed more likely to avoid attachments in the aesthetic region than younger patients (3.1 ± 3.0 points vs. 1.9 ± 2.2 points, *p* = 0.041), while younger patients were more likely to accept a prolonged treatment time to achieve the planned treatment outcome (7.5 ± 2.3 points vs. 5.8 ± 3.5 points, *p* = 0.015)

Patients with a higher initial PAR index were more worried about possible eating and drinking restrictions during CAT compared to patients with a lower PAR index (8.5 ± 1.6 points vs. 6.6 ± 3.2 points, *p* = 0.002). Further, patients with a higher initial PAR index were more worried about pain that might occur during CAT (8.8 ± 1.6 points vs. 7.0 ± 3.0 points, *p* = 0.001).

Patients with an initial OHIP simple count score ≥ 1 expected more likely to gain confidence after the treatment (7.2 ± 3.2 points vs. 5.5 ± 3.6 points, *p* = 0.041), while patients with an initial OHIP simple count score < 1 expected more likely that CAT would reduce treatment duration compared to a treatment with orthodontic brackets (5.5 ± 3.1 points vs. 3.7 ± 3.0 points, *p* = 0.016).

## Discussion

Expectation is a key psychological factor influencing patients’ evaluation of treatment quality and satisfaction with the outcome, especially in long-term treatments where aesthetic plays a significant role [29]. Patients often assess treatment effectiveness by comparing expectations with actual experiences, and discrepancies between the two can affect treatment cooperation and compliance [30, 31]. Conversely, positive expectations can enhance treatment success, as demonstrated by improved outcomes in placebo interventions [31, 32]. Hence, the present study collected extensive information on patients’ expectations regarding limitations, reasons for second opinion, treatment efficiency and predictability, willingness to compromise, and treatment quality regarding CAT.

The present data, although based on a non-validated questionnaire, clearly indicate that patients seeking CAT have very high expectations regarding treatment efficiency and outcome as well as aesthetics during and after treatment. Such high expectations of patients seeking CAT have been also stated recently based on analyses of social media posts [18, 19]. In one of these previous publications, 90% of the patients initially expressed positive expectations towards CAT. However, it was also shown in this specific publication that this might change during treatment, given that 54.8% of patients reported dissatisfaction later on; complaints centred on aesthetics, discomfort, dietary restrictions, and speech difficulties [18]. Similar, laypeople and dental patients, who were in fact not specifically pursuing orthodontic treatment, judge CAT as more favourable and more likely to use [20, 21]; either in comparison to hybrid treatments combining CAT with braces or mini-implants [20] or due to the assumed preferable aesthetics and comfort [21]. Hence, it comes without surprise, that the data herein also indicated that one of the major reasons why patients are asking for CAT is to avoid visibility of orthodontic appliances. This wish might even end in rejecting the treatment if not possible with CAT, i.e., in one of the previous surveys 62% of the patients rejected treatment with visible appliances [12]. This is further supported herein by a strong agreement of the patients to seek a second opinion in case they were told that treatment with CAT were not possible or has to be combined with orthodontic brackets.

In this context, the main motivation for orthodontic treatment in general appears to be aesthetics, as stated for example by about 70% of orthodontic patients [33]. The aesthetic-driven motivation appears even higher in CAT patients rising up to 97% [12]; for example, crowding has been mentioned in a previous study as the main reason to seek CAT [17]. This goes well along with the strong wish of patients herein to see a simulation of the expected treatment outcome prior to treatment initiation. However, patients’ motivation might not only be aesthetics and function improvement follows closely the wish for aesthetic improvement [34]. Herein, patients highly agreed that they also want to prevent potential future problems with their teeth/jaws. In 2003, a mere 3% of subjects indicated that ensuring long-term dental health constituted the primary rationale for pursuing CAT [12]. Conversely, in more recent surveys, the correction of malocclusion was the motivation to undergo orthodontic treatment for about 40 to 70% of the patients [33, 35], and a cross-sectional study suggested that adult patients with higher educational levels tend to put more importance to the oral function [36]. Patients’ expectations regarding orthodontic treatment in general appear to have increased over time and particular its effect on overall well-being. The more satisfied adult patients were with their facial appearance, the higher their expectations were on the impact of orthodontic treatment on their general well-being and their oral function [37]. However, these studies included patients undergoing both fixed orthodontic treatment and CAT, which might shift the population to a more health-oriented one.

Interestingly, many patients seeking CAT appear inflexible to change their choice of treatment modality (i.e., CAT versus fixed orthodontic treatment), and patients previously emphasized their strong preference for aligners over fixed orthodontic appliances [18, 19]. However, it appeared herein that patients still expect to a very high degree that CAT will correct their malocclusion at least as good as orthodontic brackets, and they highly expect that the treatment result will endure for their lifetime. In the present population patients appeared overall not willing to compromise on their treatment, once they have decided to undergo CAT. For example, herein patients appeared not willing to compromise on the planned treatment outcome, even if this would decrease treatment time or costs. This finding is consistent with the results of a survey conducted on parents of orthodontic patients; the parents were even more likely to consent to an extended treatment than the orthodontists themselves [38]. Nevertheless, patient satisfaction with the final treatment outcome might not vary significantly between patients achieving an ideal outcome and those with a compromised outcome [39]. But somewhat controversial results have been reported previously as well. In a study presenting pictures of clear aligners and fixed orthodontic appliances to prospective patients, 26 out of 49 subjects indicated a preference for the clear aligners [40]. However, when confronted with the higher costs and a possible inferior outcome with aligners, only 15 out of 49 patients would still prefer aligners [40].

The expected limitations during treatment appear somewhat similar, independent whether patients think about CAT or fixed orthodontic treatment, and patients seeking CAT are still aware of regular limitations occurring during treatment. Herein, patients strongly assumed pain and restrictions with eating and drinking habits to occur during treatment. This is well comparable to the expectations of patients regarding fixed appliances, which included moderate pain levels, restrictions concerning diet and daily living, and minimal restrictions on leisure and social activities [41]. In this context, patients expect to be well informed about the treatment process [42], such as treatment duration, potential tooth extraction, and possible pain or discomfort regarding eating, speaking, and oral hygiene. This was reflected herein, with a strong wish of the patients to see a simulation of the expected treatment outcome prior to starting the treatment, while a previous publication could also show, that a lack of such information can indeed impair patients’ satisfaction with the treatment [43].

Within the exploratory analysis on differences due to gender, age, PAR index, and OHIP simple count score we found a few interesting aspects. Herein, female participants had higher expectations on the predictability and time efficacy of CAT, while male participants were more likely to decline the use of elastics. Other previously reported gender-related differences were for example a stronger social motivation for treatment among males than females, while females mentioned more frequently temporomandibular joint concerns [44]. Further, in the present study younger patients appeared more likely to accept a prolonged treatment time to achieve the planned treatment outcome, which is consistent with previously documented higher compliance rates among younger adults [45]. Finally, patients with a higher PAR index, indicating a more severe malocclusion, reported herein greater concerns regarding potential limitations during eating and drinking and were more worried about possible discomfort during CAT. However, it was shown previously that patients who are aware of the severity of their treatment needs appear also more likely to tolerate orthodontic appliances and finally appear to experience less pain [46]. However, when interpreting these aspects, it should be kept in mind that the data herein are based on an exploratory analysis without correction for multiple testing. In this context the present study has some limitations, which should be considered when interpreting the results. While the questionnaire on OHRQoL is a validated and well accepted questionnaire [47], the statements regarding patients’ opinion on CAT have been developed within a group of experienced orthodontists. This questionnaire has not been used previously and has not been validated. The decision not to specifically validate the questionnaire was taken, as it is not intended to use the questionnaire for calculating an index or score. Nevertheless, this should be considered when interpreting the results, i.e., the survey is providing an overview on clinically relevant questions but does not represent a validated tool. In addition, the statistical analysis to assess any differences in patients’ opinion due to age, gender, PAR index, and/or OHIP simple count score should be considered as exploratory and – as stated above – no corrections for multiple testing have been performed. Further, the age of the population ranged from 18 to 63 years, and three quarters of the population have been female. Hence, the answers might be representative for adults, but not for adolescents. The unequal gender representation might be considered as a limitation as well, but on the other side, it represents the regular population seeking CAT. Specifically, the patient demographics (i.e., age and gender) correspond well with previous studies investigating patients undergoing CAT. Often a mean/median age of 30 to 35 years and a high percentage of female patients (i.e., 68 to 78%) are reported [12, 17, 22]. In addition, this high percentage of female patients is not limited to CAT, for example, an online survey among adult orthodontic patients treated with different orthodontic appliances presented with an even higher percentage of women, i.e., almost 88% [33]. Finally, no control group including patients choosing fixed orthodontic appliances was included, which would have allowed to assess any potential differences regarding patients’ needs, expectations, and concerns regarding treatment with either fixed orthodontic appliances or CAT.

## Conclusion

This is the first part of a prospective cohort study collecting patients’ expectations regarding CAT. The data collected prior to initiation of CAT provided the following interesting clinical aspects:

- Patients seeking CAT appear convinced about their treatment choice and suggesting a different treatment modality might cause the patient to look for a second opinion.
- Patients seeking CAT appear often aesthetically driven. They probably want to avoid visibility of orthodontic brackets and often wish to see a treatment simulation prior to treatment initiation. A second frequent motivation to seek CAT was avoidance of future oral health problems.
- Patients seeking CAT appear reluctant to compromise on the treatment outcome when choosing CAT over fixed orthodontics. They express a desire for a high degree of predictability in the treatment plan and a long-lasting result. In addition, they seem not willing to deviate from the planned treatment outcome, even if this would reduce costs, treatment time, and/or the need of attachments in the aesthetic region.
- Patients seeking CAT appear to be aware of regularly occurring limitations, such as pain and restrictions in eating and drinking habits, but they still expect a certain level of comfort due to choosing CAT.
- Patients seeking CAT often expect their dentist being present during most of the appointments

Knowledge about these expectations can support our patient communication and thereby improve patients’ satisfaction with the treatment outcome.

## Supplementary Information

Below is the link to the electronic supplementary material.

Supplementary Material 1

## Data Availability

The datasets generated and analysed during the current study are not publicly available due to their highly sensitive nature, but tables with raw data are available from the corresponding author upon reasonable request.

## Abbreviations

CAT: clear aligner therapy
OHIP: Oral Health Impact Profile
OHRQoL: oral health related quality of life
PAR: Peer Assessment Rating
VAS: visual analogue scale
