# Supplementary material for: Patients’ needs, expectations & concerns regarding clear aligner treatment
Source: Clin Oral Investig. 2026 May 29;30(6):256. doi: 10.1007/s00784-026-06903-x (PMC13219153; doi:10.1007/s00784-026-06903-x)
Supplement: Supplementary file 1 — Supplementary Material 1 [file 784_2026_6903_MOESM1_ESM.docx]

Supplementary Material

The supplementary material includes five tables presenting an exploratory subgroup analysis examining differences in patients’ responses based on sex, age, initial PAR index, and OHIP simple count score.

**Table 1S.** Subgroup analysis of patients’ expectations about the limitations of CAT (Category 1).

| **Question** | **Category** | | **n** | **Mean** | **SD** | **p-value** |
| --- | --- | --- | --- | --- | --- | --- |
| Please indicate how important the following topic is for you: Avoidance of visibility of orthodontic brackets on the outer surface of the teeth | Sex | male | 21 | 7.8 | 2.8 | 0.952 |
|  |  | female | 59 | 7.9 | 2.8 |  |
|  | Age | ≤ 35 | 46 | 8.2 | 2.5 | 0.252 |
|  |  | > 35 | 34 | 7.4 | 3.0 |  |
|  | PAR-Index | ≤ 17 | 42 | 7.9 | 3.0 | 0.888 |
|  |  | > 17 | 36 | 8.0 | 2.4 |  |
|  | OHIP-Score | < 1 | 34 | 7.8 | 3.0 | 0.835 |
|  |  | ≥ 1 | 46 | 7.9 | 2.6 |  |
|  | | | | | | |
| I expect pain might occur during tooth movement. | Sex | male | 21 | 7.9 | 1.8 | 0.649 |
|  |  | female | 59 | 7.7 | 2.9 |  |
|  | Age | ≤ 35 | 46 | 8.1 | 2.2 | 0.129 |
|  |  | > 35 | 34 | 7.2 | 3.1 |  |
|  | PAR-Index | ≤ 17 | 42 | 7.0 | 3.0 | **0.001** |
|  |  | > 17 | 36 | 8.8 | 1.6 |  |
|  | OHIP-Score | < 1 | 34 | 7.5 | 2.7 | 0.476 |
|  |  | ≥ 1 | 46 | 7.9 | 2.6 |  |
|  | | | | | | |
| I expect that my eating and drinking habits will be restricted during CAT. | Sex | male | 21 | 6.6 | 3.0 | 0.177 |
|  |  | female | 59 | 7.6 | 2.7 |  |
|  | Age | ≤ 35 | 46 | 7.1 | 3.0 | 0.420 |
|  |  | > 35 | 34 | 7.6 | 2.6 |  |
|  | PAR-Index | ≤ 17 | 42 | 6.6 | 3.2 | **0.002** |
|  |  | > 17 | 36 | 8.5 | 1.6 |  |
|  | OHIP-Score | < 1 | 34 | 7.2 | 2.8 | 0.760 |
|  |  | ≥ 1 | 46 | 7.4 | 2.9 |  |
|  | | | | | | |
| I expect that the aligner will be comfortable to wear during my treatment. | Sex | male | 21 | 6.0 | 2.5 | 0.080 |
|  |  | female | 59 | 7.2 | 2.7 |  |
|  | Age | ≤ 35 | 46 | 6.4 | 2.8 | 0.087 |
|  |  | > 35 | 34 | 7.5 | 2.4 |  |
|  | PAR-Index | ≤ 17 | 42 | 7.2 | 2.5 | 0.154 |
|  |  | > 17 | 36 | 6.3 | 2.9 |  |
|  | OHIP-Score | < 1 | 34 | 6.8 | 2.6 | 0.727 |
|  |  | ≥ 1 | 46 | 7.0 | 2.8 |  |
|  | | | | | | |
| I expect my pronunciation to deteriorate during treatment. | Sex | male | 21 | 6.1 | 2.9 | 0.758 |
|  |  | female | 59 | 5.8 | 3.0 |  |
|  | Age (years) | ≤ 35 | 46 | 5.9 | 3.2 | 0.910 |
|  |  | > 35 | 34 | 5.9 | 2.7 |  |
|  | PAR-Index | ≤ 17 | 42 | 6.1 | 3.0 | 0.709 |
|  |  | > 17 | 36 | 5.9 | 2.8 |  |
|  | OHIP-Score | < 1 | 34 | 5.5 | 3.0 | 0.281 |
|  |  | ≥ 1 | 46 | 6.2 | 2.9 |  |
|  | | | | | | |
| I expect that CAT will be perceived by other people. | Sex | male | 21 | 6.3 | 2.5 | 0.259 |
|  |  | female | 59 | 5.5 | 3.3 |  |
|  | Age | ≤ 35 | 46 | 5.4 | 3.1 | 0.194 |
|  |  | > 35 | 34 | 6.3 | 3.2 |  |
|  | PAR-Index | ≤ 17 | 42 | 5.3 | 3.5 | 0.104 |
|  |  | > 17 | 36 | 6.5 | 2.5 |  |
|  | OHIP-Score | < 1 | 34 | 6.0 | 3.0 | 0.520 |
|  |  | ≥ 1 | 46 | 5.6 | 3.3 |  |
|  | | | | | | |
| I expect that the attachments (buttons glued to the tooth surface) will restrict me. | Sex | male | 21 | 4.3 | 2.8 | 0.749 |
|  |  | female | 59 | 4.0 | 3.2 |  |
|  | Age | ≤ 35 | 46 | 4.3 | 3.1 | 0.527 |
|  |  | > 35 | 34 | 3.8 | 3.1 |  |
|  | PAR-Index | ≤ 17 | 42 | 3.7 | 3.2 | 0.203 |
|  |  | > 17 | 36 | 4.6 | 3.0 |  |
|  | OHIP-Score | < 1 | 34 | 4.2 | 3.3 | 0.812 |
|  |  | ≥ 1 | 46 | 4.0 | 3.0 |  |
|  | | | | | | |
| I expect that my access for oral hygiene will be restricted during the treatment. | Sex | male | 21 | 3.8 | 2.7 | 0.189 |
|  |  | female | 59 | 2.9 | 2.9 |  |
|  | Age | ≤ 35 | 46 | 3.2 | 2.9 | 0.834 |
|  |  | > 35 | 34 | 3.0 | 3.0 |  |
|  | PAR-Index | ≤ 17 | 42 | 2.7 | 2.8 | 0.231 |
|  |  | > 17 | 36 | 3.5 | 3.1 |  |
|  | OHIP-Score | < 1 | 34 | 2.8 | 2.9 | 0.382 |
|  |  | ≥ 1 | 46 | 3.4 | 2.9 |  |
|  | | | | | | |
| I expect my self-confidence to decrease during treatment. | Sex | male | 21 | 2.7 | 2.2 | 0.432 |
|  |  | female | 59 | 2.2 | 2.7 |  |
|  | Age | ≤ 35 | 46 | 2.4 | 2.7 | 0.935 |
|  |  | > 35 | 34 | 2.3 | 2.4 |  |
|  | PAR-Index | ≤ 17 | 42 | 2.1 | 2.7 | 0.329 |
|  |  | > 17 | 36 | 2.7 | 2.4 |  |
|  | OHIP-Score | < 1 | 34 | 1.9 | 2.5 | 0.221 |
|  |  | ≥ 1 | 46 | 2.6 | 2.6 |  |

*CAT, clear aligner therapy; n, number; OHIP, Oral Health Impact Profile; PAR, peer assessment rating; SD, standard deviation.*

*Bold values indicate a statistically significant p-value < 0.05.*

*If the sum of the various categories does not result in 82, the remaining values are missing.*

**Table 2S.** Subgroup analysis of patients’ expectations regarding seeking a second orthodontic opinion for CAT (Category 2).

| **Question** | **Category** | | **n** | **Mean** | **SD** | **p-value** |
| --- | --- | --- | --- | --- | --- | --- |
| I would seek a second opinion if I was told that treatment with CAT is not possible. | Sex | male | 21 | 7.0 | 3.2 | 0.726 |
|  |  | female | 59 | 7.2 | 3.4 |  |
|  | Age | ≤ 35 | 46 | 6.9 | 3.5 | 0.396 |
|  |  | > 35 | 34 | 7.5 | 3.2 |  |
|  | PAR-Index | ≤ 17 | 42 | 6.7 | 3.4 | 0.266 |
|  |  | > 17 | 36 | 7.6 | 3.3 |  |
|  | OHIP-Score | < 1 | 34 | 7.0 | 3.1 | 0.722 |
|  |  | ≥ 1 | 46 | 7.3 | 3.5 |  |
|  | | | | | | |
| I would seek a second opinion if I was told that due to a significant lack of space the extraction of one tooth / several teeth is necessary. | Sex | male | 21 | 6.6 | 3.4 | 0.873 |
|  |  | female | 59 | 6.7 | 3.6 |  |
|  | Age | ≤ 35 | 46 | 6.7 | 3.7 | 0.953 |
|  |  | > 35 | 34 | 6.7 | 3.5 |  |
|  | PAR-Index | ≤ 17 | 42 | 6.8 | 3.6 | 0.662 |
|  |  | > 17 | 36 | 6.4 | 3.7 |  |
|  | OHIP-Score | < 1 | 34 | 6.9 | 3.5 | 0.575 |
|  |  | ≥ 1 | 46 | 6.5 | 3.7 |  |
|  | | | | | | |
| I would seek a second opinion if I was told that treatment with CAT is only possible in combination with orthodontic brackets. | Sex | male | 21 | 5.3 | 3.3 | 0.242 |
|  |  | female | 59 | 6.3 | 3.8 |  |
|  | Age | ≤ 35 | 46 | 6.1 | 3.6 | 0.980 |
|  |  | > 35 | 34 | 6.1 | 3.8 |  |
|  | PAR-Index | ≤ 17 | 42 | 5.7 | 3.7 | 0.475 |
|  |  | > 17 | 36 | 6.3 | 3.7 |  |
|  | OHIP-Score | < 1 | 34 | 5.6 | 3.5 | 0.346 |
|  |  | ≥ 1 | 46 | 6.4 | 3.8 |  |
|  | | | | | | |
| I would seek a second opinion if I was told that treatment is only possible in combination with polishing the teeth laterally, i.e., with some removal of the tooth substance. | Sex | male | 21 | 5.2 | 3.2 | 0.944 |
|  |  | female | 59 | 5.1 | 3.8 |  |
|  | Age | ≤ 35 | 46 | 5.2 | 3.5 | 0.919 |
|  |  | > 35 | 34 | 5.1 | 3.9 |  |
|  | PAR-Index | ≤ 17 | 42 | 4.8 | 3.5 | 0.376 |
|  |  | > 17 | 36 | 5.5 | 3.8 |  |
|  | OHIP-Score | < 1 | 34 | 5.3 | 3.6 | 0.827 |
|  |  | ≥ 1 | 46 | 5.1 | 3.7 |  |
|  | | | | | | |
| I would seek a second opinion if I was told that treatment with CAT is only possible in combination with the use of elastic bands. | Sex | male | 21 | 4.5 | 3.4 | 0.723 |
|  |  | female | 59 | 4.9 | 4.0 |  |
|  | Age | ≤ 35 | 46 | 4.5 | 3.8 | 0.429 |
|  |  | > 35 | 34 | 5.2 | 4.0 |  |
|  | PAR-Index | ≤ 17 | 42 | 4.9 | 3.9 | 0.717 |
|  |  | > 17 | 36 | 4.6 | 3.8 |  |
|  | OHIP-Score | < 1 | 34 | 5.6 | 3.5 | 0.660 |
|  |  | ≥ 1 | 46 | 6.4 | 3.8 |  |

*CAT, clear aligner therapy; n, number; OHIP, Oral Health Impact Profile; PAR, peer assessment rating; SD, standard deviation.*

*Bold values indicate a statistically significant p-value < 0.05.*

*If the sum of the various categories does not result in 82, the remaining values are missing.*

**Table 3S.** Subgroup analysis of patients’ expectations regarding efficiency and predictability of CAT (Category 3).

| **Question** | **Category** | | **n** | **Mean** | **SD** | **p-value** |
| --- | --- | --- | --- | --- | --- | --- |
| Please indicate how important the following topic is for you: Prevention of potential future problems with the teeth/jaws | Sex | male | 21 | 9.2 | 0.7 | 0.718 |
|  |  | female | 59 | 9.2 | 1.7 |  |
|  | Age | ≤ 35 | 46 | 8.9 | 1.8 | 0.122 |
|  |  | > 35 | 34 | 9.4 | 0.8 |  |
|  | PAR-Index | ≤ 17 | 42 | 9.4 | 0.9 | 0.117 |
|  |  | > 17 | 36 | 8.8 | 1.9 |  |
|  | OHIP-Score | < 1 | 34 | 9.1 | 1.2 | 0.881 |
|  |  | ≥ 1 | 46 | 9.1 | 1.6 |  |
|  | | | | | | |
| Please indicate how important the following topic is for you: Predictability of treatment success | Sex | male | 21 | 7.7 | 2.4 | **0.037** |
|  |  | female | 59 | 8.9 | 1.6 |  |
|  | Age | ≤ 35 | 46 | 8.3 | 2.2 | 0.057 |
|  |  | > 35 | 34 | 9.0 | 1.3 |  |
|  | PAR-Index | ≤ 17 | 42 | 8.6 | 2.0 | 0.961 |
|  |  | > 17 | 36 | 8.6 | 1.8 |  |
|  | OHIP-Score | < 1 | 34 | 8.5 | 2.0 | 0.753 |
|  |  | ≥ 1 | 46 | 8.6 | 1.8 |  |
|  | | | | | | |
| Please indicate how important the following topic is for you: Time efficiency of treatment | Sex | male | 21 | 6.8 | 2.2 | **0.036** |
|  |  | female | 59 | 8.1 | 2.3 |  |
|  | Age | ≤ 35 | 46 | 7.1 | 2.5 | **0.007** |
|  |  | > 35 | 34 | 8.5 | 1.8 |  |
|  | PAR-Index | ≤ 17 | 42 | 7.5 | 2.6 | 0.402 |
|  |  | > 17 | 36 | 7.9 | 2.2 |  |
|  | OHIP-Score | < 1 | 34 | 7.9 | 2.2 | 0.534 |
|  |  | ≥ 1 | 46 | 7.6 | 2.4 |  |
|  | | | | | | |
| It is important for me to see a simulation of the expected treatment outcome prior to starting the treatment. | Sex | male | 21 | 6.5 | 3.3 | 0.086 |
|  |  | female | 59 | 7.9 | 2.7 |  |
|  | Age | ≤ 35 | 46 | 7.6 | 2.6 | 0.837 |
|  |  | > 35 | 34 | 7.4 | 3.3 |  |
|  | PAR-Index | ≤ 17 | 42 | 7.5 | 3.1 | 0.743 |
|  |  | > 17 | 36 | 7.7 | 2.6 |  |
|  | OHIP-Score | < 1 | 34 | 7.6 | 2.6 | 0.951 |
|  |  | ≥ 1 | 46 | 7.5 | 3.1 |  |
|  | | | | | | |
| Please indicate how important the following topic is for you: Cost-effectiveness of the treatment | Sex | male | 21 | 6.7 | 2.6 | 0.360 |
|  |  | female | 59 | 7.3 | 2.7 |  |
|  | Age | ≤ 35 | 46 | 6.8 | 2.8 | 0.137 |
|  |  | > 35 | 34 | 7.7 | 2.4 |  |
|  | PAR-Index | ≤ 17 | 42 | 7.3 | 2.4 | 0.464 |
|  |  | > 17 | 36 | 6.9 | 3.0 |  |
|  | OHIP-Score | < 1 | 34 | 7.2 | 2.5 | 0.831 |
|  |  | ≥ 1 | 46 | 7.1 | 2.8 |  |
|  | | | | | | |
| I expect treatment with CAT to take less time than with orthodontic brackets. | Sex | male | 21 | 4.2 | 3.0 | 0.623 |
|  |  | female | 59 | 4.6 | 3.3 |  |
|  | Age | ≤ 35 | 46 | 4.3 | 3.2 | 0.509 |
|  |  | > 35 | 34 | 4.7 | 3.2 |  |
|  | PAR-Index | ≤ 17 | 42 | 4.3 | 3.4 | 0.635 |
|  |  | > 17 | 36 | 4.7 | 3.1 |  |
|  | OHIP-Score | < 1 | 34 | 5.5 | 3.1 | **0.016** |
|  |  | ≥ 1 | 46 | 3.1 | 3.0 |  |

*CAT, clear aligner therapy; n, number; OHIP, Oral Health Impact Profile; PAR, peer assessment rating; SD, standard deviation.*

*Bold values indicate a statistically significant p-value < 0.05.*

*If the sum of the various categories does not result in 82, the remaining values are missing.*

**Table 4S.** Subgroup analysis of patients’ expectations regarding willingness to compromise with CAT (Category 4).

| **Question** | **Category** | | **n** | **Mean** | **SD** | **p-value** |
| --- | --- | --- | --- | --- | --- | --- |
| I would deny the use of elastic bands, even if this would result in a certain failure to achieve the previously planned correction of the tooth position. | Sex | male | 21 | 3.7 | 2.9 | **0.034** |
|  |  | female | 59 | 2.2 | 2.7 |  |
|  | Age | ≤ 35 | 46 | 2.2 | 2.5 | 0.181 |
|  |  | > 35 | 34 | 3.1 | 3.2 |  |
|  | PAR-Index | ≤ 17 | 42 | 2.5 | 2.7 | 0.980 |
|  |  | > 17 | 36 | 2.5 | 2.9 |  |
|  | OHIP-Score | < 1 | 34 | 2.8 | 2.7 | 0.584 |
|  |  | ≥ 1 | 46 | 2.4 | 2.9 |  |
|  | | | | | | |
| I would like to avoid gluing of attachments (buttons glued to the tooth surface) on the anterior teeth, even if this would limit the possibilities for tooth movement. | Sex | male | 21 | 2.9 | 2.8 | 0.308 |
|  |  | female | 59 | 2.2 | 2.6 |  |
|  | Age | ≤ 35 | 46 | 1.9 | 2.2 | **0.041** |
|  |  | > 35 | 34 | 3.1 | 3.0 |  |
|  | PAR-Index | ≤ 17 | 42 | 2.0 | 2.4 | 0.196 |
|  |  | > 17 | 36 | 2.7 | 2.7 |  |
|  | OHIP-Score | < 1 | 34 | 2.4 | 2.1 | 0.878 |
|  |  | ≥ 1 | 46 | 2.4 | 3.0 |  |
|  | | | | | | |
| I would accept a slightly inferior improvement of the tooth position if this would reduce treatment costs. | Sex | male | 21 | 2.8 | 2.5 | 0.402 |
|  |  | female | 59 | 2.3 | 2.5 |  |
|  | Age | ≤ 35 | 46 | 2.3 | 2.4 | 0.674 |
|  |  | > 35 | 34 | 2.6 | 2.7 |  |
|  | PAR-Index | ≤ 17 | 42 | 2.0 | 2.2 | 0.161 |
|  |  | > 17 | 36 | 2.8 | 2.8 |  |
|  | OHIP-Score | < 1 | 34 | 2.6 | 2.3 | 0.635 |
|  |  | ≥ 1 | 46 | 2.3 | 2.7 |  |
|  | | | | | | |
| I would accept a slightly inferior improvement of the tooth position if this would reduce the treatment time. | Sex | male | 21 | 2.3 | 2.1 | 0.648 |
|  |  | female | 59 | 2.0 | 2.4 |  |
|  | Age | ≤ 35 | 46 | 1.8 | 2.1 | 0.293 |
|  |  | > 35 | 34 | 2.4 | 2.6 |  |
|  | PAR-Index | ≤ 17 | 42 | 2.1 | 2.3 | 0.774 |
|  |  | > 17 | 36 | 2.0 | 2.4 |  |
|  | OHIP-Score | < 1 | 34 | 2.3 | 2.3 | 0.454 |
|  |  | ≥ 1 | 46 | 1.9 | 2.3 |  |

*n, number; OHIP, Oral Health Impact Profile; PAR, peer assessment rating; SD, standard deviation.*

*Bold values indicate a statistically significant p-value < 0.05.*

*If the sum of the various categories does not result in 82, the remaining values are missing.*

**Table 5S.** Subgroup analysis of patients’ expectations regarding quality of treatment with CAT (Category 5).

| **Question** | **Category** | | **n** | **Mean** | **SD** | **p-value** |
| --- | --- | --- | --- | --- | --- | --- |
| I expect CAT to correct my malocclusion at least as good as orthodontic brackets. | Sex | male | 21 | 9.2 | 0.9 | 0.187 |
|  |  | female | 59 | 9.5 | 0.8 |  |
|  | Age | ≤ 35 | 46 | 9.5 | 0.8 | 0.514 |
|  |  | > 35 | 34 | 9.4 | 1.0 |  |
|  | PAR-Index | ≤ 17 | 42 | 9.4 | 0.9 | 0.569 |
|  |  | > 17 | 36 | 9.5 | 0.8 |  |
|  | OHIP-Score | < 1 | 34 | 9.4 | 0.8 | 0.850 |
|  |  | ≥ 1 | 46 | 9.5 | 0.9 |  |
|  | | | | | | |
| I expect the treatment outcome to last for a lifetime. | Sex | male | 21 | 8.3 | 2.4 | 0.280 |
|  |  | female | 59 | 8.9 | 2.0 |  |
|  | Age | ≤ 35 | 46 | 8.7 | 2.1 | 0.934 |
|  |  | > 35 | 34 | 8.8 | 2.2 |  |
|  | PAR-Index | ≤ 17 | 42 | 8.7 | 2.5 | 0.648 |
|  |  | > 17 | 36 | 8.9 | 1.6 |  |
|  | OHIP-Score | < 1 | 34 | 8.7 | 2.1 | 0.870 |
|  |  | ≥ 1 | 46 | 8.8 | 2.1 |  |
|  | | | | | | |
| I expect the treatment outcome to last at least 5 years. | Sex | male | 21 | 8.7 | 2.8 | 0.937 |
|  |  | female | 59 | 8.6 | 2.9 |  |
|  | Age | ≤ 35 | 46 | 9.1 | 2.3 | 0.137 |
|  |  | > 35 | 34 | 8.1 | 3.4 |  |
|  | PAR-Index | ≤ 17 | 42 | 8.6 | 2.9 | 0.375 |
|  |  | > 17 | 36 | 9.1 | 2.2 |  |
|  | OHIP-Score | < 1 | 34 | 8.9 | 2.5 | 0.506 |
|  |  | ≥ 1 | 46 | 8.5 | 3.1 |  |
|  | | | | | | |
| I expect my dentist/orthodontist to be personally present at each appointment during my CAT. | Sex | male | 21 | 6.7 | 3.0 | 0.650 |
|  |  | female | 59 | 7.0 | 3.1 |  |
|  | Age | ≤ 35 | 46 | 7.0 | 2.9 | 0.979 |
|  |  | > 35 | 34 | 6.9 | 3.2 |  |
|  | PAR-Index | ≤ 17 | 42 | 7.3 | 3.0 | 0.376 |
|  |  | > 17 | 36 | 6.7 | 2.9 |  |
|  | OHIP-Score | < 1 | 34 | 7.6 | 2.3 | 0.102 |
|  |  | ≥ 1 | 46 | 6.5 | 3.4 |  |
|  | | | | | | |
| I would accept a prolonged treatment time including additional aligners if the planned treatment outcome has not been fully achieved. | Sex | male | 21 | 7.0 | 2.5 | 0.649 |
|  |  | female | 59 | 6.7 | 3.1 |  |
|  | Age | ≤ 35 | 46 | 7.5 | 2.3 | **0.015** |
|  |  | > 35 | 34 | 5.8 | 3.5 |  |
|  | PAR-Index | ≤ 17 | 42 | 6.8 | 3.0 | 0.857 |
|  |  | > 17 | 36 | 6.9 | 2.9 |  |
|  | OHIP-Score | < 1 | 34 | 6.7 | 2.6 | 0.830 |
|  |  | ≥ 1 | 46 | 6.8 | 3.2 |  |
|  | | | | | | |
| I expect to gain confidence after the treatment. | Sex | male | 21 | 5.5 | 3.7 | 0.161 |
|  |  | female | 59 | 6.8 | 3.3 |  |
|  | Age | ≤ 35 | 46 | 7.0 | 3.3 | 0.120 |
|  |  | > 35 | 34 | 5.8 | 3.6 |  |
|  | PAR-Index | ≤ 17 | 42 | 6.1 | 3.7 | 0.360 |
|  |  | > 17 | 36 | 6.8 | 3.1 |  |
|  | OHIP-Score | < 1 | 34 | 5.5 | 3.6 | **0.041** |
|  |  | ≥ 1 | 46 | 7.2 | 3.2 |  |
|  | | | | | | |
| I expect CAT to correct my malocclusion better than orthodontic brackets. | Sex | male | 20 | 4.5 | 2.6 | **0.022** |
|  |  | female | 58 | 6.2 | 3.4 |  |
|  | Age | ≤ 35 | 44 | 5.4 | 3.3 | 0.200 |
|  |  | > 35 | 34 | 6.3 | 3.1 |  |
|  | PAR-Index | ≤ 17 | 42 | 5.3 | 3.4 | 0.172 |
|  |  | > 17 | 35 | 6.3 | 3.0 |  |
|  | OHIP-Score | < 1 | 34 | 6.3 | 3.1 | 0.223 |
|  |  | ≥ 1 | 46 | 5.4 | 3.4 |  |
|  | | | | | | |
| I expect better job opportunities due to CAT. | Sex | male | 21 | 2.2 | 2.3 | 0.617 |
|  |  | female | 59 | 2.5 | 2.5 |  |
|  | Age | ≤ 35 | 46 | 2.6 | 2.6 | 0.426 |
|  |  | > 35 | 34 | 2.1 | 2.2 |  |
|  | PAR-Index | ≤ 17 | 42 | 2.4 | 2.5 | 0.857 |
|  |  | > 17 | 36 | 2.5 | 2.4 |  |
|  | OHIP-Score | < 1 | 34 | 2.6 | 2.4 | 0.587 |
|  |  | ≥ 1 | 46 | 2.4 | 2.5 |  |

*CAT, clear aligner therapy; n, number; OHIP, Oral Health Impact Profile; PAR, peer assessment rating; SD, standard deviation.*

*Bold values indicate a statistically significant p-value < 0.05.*

*If the sum of the various categories does not result in 82, the remaining values are missing.*
